# damidseq_pipeline: an automated pipeline for processing DamID sequencing datasets

**DOI:** 10.1093/bioinformatics/btv386

**Published:** 2015-06-25

**Authors:** Owen J. Marshall, Andrea H. Brand

**Affiliations:** Wellcome Trust/Cancer Research UK Gurdon Institute, Cambridge, CB2 1QN, UK

## Abstract

**Summary:** DamID is a powerful technique for identifying regions of the genome bound by a DNA-binding (or DNA-associated) protein. Currently, no method exists for automatically processing next-generation sequencing DamID (DamID-seq) data, and the use of DamID-seq datasets with normalization based on read-counts alone can lead to high background and the loss of bound signal. DamID-seq thus presents novel challenges in terms of normalization and background minimization. We describe here damidseq_pipeline, a software pipeline that performs automatic normalization and background reduction on multiple DamID-seq FASTQ datasets.

**Availability and implementation:** Open-source and freely available from http://owenjm.github.io/damidseq_pipeline. The damidseq_pipeline is implemented in Perl and is compatible with any Unix-based operating system (e.g. Linux, Mac OSX).

**Contact:** o.marshall@gurdon.cam.ac.uk

**Supplementary information:** Supplementary data are available at *Bioinformatics* online.

## 1 Introduction

DamID is a well-established technique for discovering regions of DNA bound by or associated with proteins (van Steensel and Henikoff, 2000). It has been used to map the genome-wide binding of transcription factors, chromatin proteins, nuclear complexes associated with DNA and RNA pol II (for e.g. Choksi *et* *al.*, 2006; Filion *et* *al.*, 2010; Singer *et al.*, 2014; Southall *et* *al.*, 2013). The technique can be performed in cell culture, whole organisms (van Steensel and Henikoff, 2000) or with cell-type specificity (Southall *et* *al.*, 2013), and requires no fixation or antibody purification.

DamID involves the fusion of a bacterial DNA adenine methylase (Dam) to any DNA-associated protein of interest. The bacterial Dam protein methylates adenine in the sequence GATC and, given that higher eukaryotes lack native adenine methylation, the DNA-binding footprint of the protein of interest is uniquely detectable through isolating sequences flanked by methylated GATC sites. However, a major consideration with DamID is that any Dam protein within the nucleus will non-specifically methylate adenines in GATC sequences at accessible regions of the genome. For this reason, DamID is always performed concurrently with a Dam-only control, and the final DNA-binding profile is typically presented as a log_2_(Dam-fusion/Dam-only) ratio.

Although the majority of published DamID experiments have used tiling microarrays for data analysis, next-generation sequencing (NGS) allows greater sensitivity and higher accuracy. Although several recent studies have used NGS with DamID (Carl and Russell, 2015; Clough *et al.*, 2014; Lie-A-Ling *et al.*, 2014; Wu and Yao, 2013), these have relied upon a comparison of peak binding intensities between read-count-normalized Dam-fusion and Dam samples. Depending on the characteristics of the Dam-fusion protein (see later) this approach may lead to real signal being lost, and correct normalization of the datasets is required to detect all binding by many Dam-fusion proteins. Here, we describe a software pipeline for the automated processing of DamID-sequencing (DamID-seq) data, including normalization and background reduction algorithms.

## 2 Algorithms

**Fig. 1.**
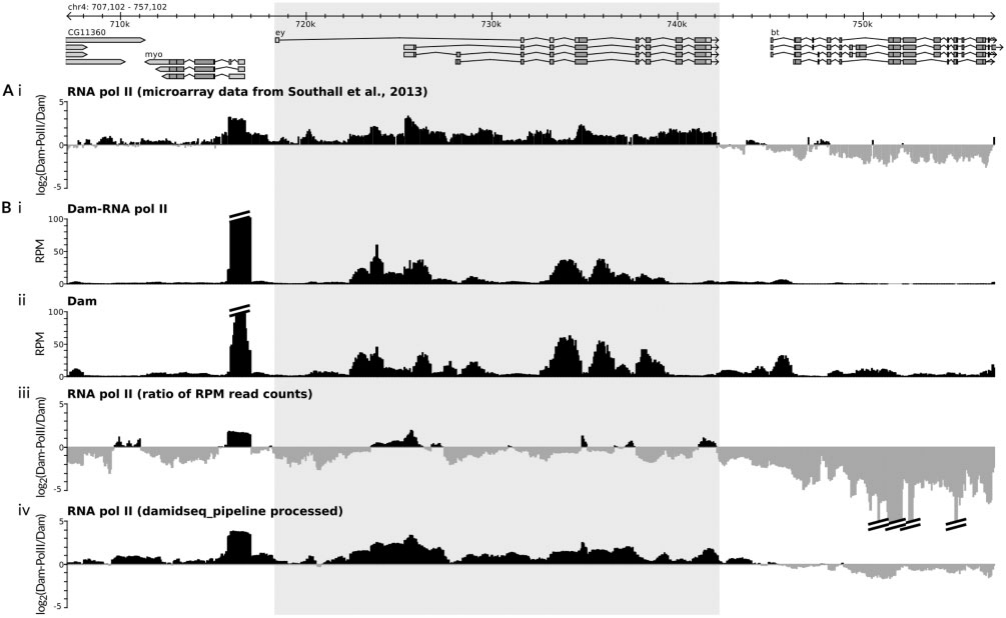
Results of the damidseq_pipeline. (**A**) The gene *eyeless (ey)* (highlighted) is expressed in *D. melanogaster* laval neural stem cells (Southall *et al.*, 2013) and previously published microarray DamID in these cells (i) shows RNA polymerase II occupancy (Southall *et al.*, 2013). (**B**) Performing DamID-seq in the same cell type illustrates the high correlation between Dam-Pol II (i) and Dam alone (ii) in terms of RPM (read counts/million mapped reads). Taking the ratio of the two RPM-normalized datasets fails to show significant RNA pol II occupancy at *ey* (iii); however, processing via the damidseq_pipeline software successfully recovers the RNA pol II occupancy profile while minimizing background (iv). See Supplementary Methods for experimental details

Although DamID-seq data can be aligned and binned as per all NGS data, two issues arise that are specific to DamID. The first major consideration is the correct normalization of the Dam-fusion and Dam-control samples. The greatest contribution to many Dam-fusion protein datasets is the non-specific methylation of accessible genomic regions (e.g. Fig. 1B), with a mean correlation between Dam alone and Dam-fusion datasets of 0.70 (*n* = 4, Spearman’s correlation). Representing the data as a (Dam-fusion/Dam) ratio in theory negates such non-specific methylation. However, strong methylation signals at highly bound regions in the Dam-fusion dataset will reduce the relative numbers of reads present at accessible genomic regions in this dataset (see, for example, the occupancy of Dam-RNA Pol II over the *eyeless* gene in Fig. 1), and normalizing the data based on read counts alone can therefore produce a strong negative bias to the ratio file [Fig. 1B (iii), Supplementary Fig. S5A]. Depending on the characteristics of the fusion protein, this negative bias can lead to real signal being lost (Fig. 1). Although microarray data inadvertently overcame this bias through the manual adjustment of laser intensities during microarray scanning, until now no method has existed for correctly normalizing DamID-seq datasets.

In order to correct for this negative bias we use the read counts from accessible genomic regions—as determined from the Dam-only dataset—as the basis for normalization, while avoiding regions likely to contain real signal in the Dam-fusion sample. We use the following algorithm to adjust the Dam-fusion dataset.

1. Given the GATC-site resolution of DamID, we divide the read counts into GATC fragments.
2. All GATC fragments lacking read counts are excluded. The remaining GATC fragments are divided into deciles.
3. Given the high probability that the highest 10% of Dam-fusion read counts represent bound signal rather than background signal, we exclude fragments that have scores in this decile.
4. The first three deciles of the Dam sample can generate inconsistent normalization values if included (Supplementary Table S2), so we exclude fragments that lie within this range.
5. The distribution of the log_2_(Dam-fusion/Dam) ratio $\left(x_{1},\;x_{2},\ldots ,\;x_{n}\right)$ for all remaining fragments is determined via the Gaussian kernel density estimate ${\hat{f}}_{h}(x)=\frac{1}{nh}\sum_{i=1}^{n}\frac{1}{\sqrt{2\pi }}\exp\left(-\frac{{\left(x_{i}-x\right)}^{2}}{2h^{2}}\right)$, where *h* is the bandwidth, estimated via the method of Silverman (1986): $h=0.9\frac{\min\left(\sigma ,\text{IQR}\right)}{1.34}n^{-1/5}$ (where *σ* is the standard deviation of the sample and IQR the interquartile range). For speed considerations, we estimate kernel density over 300 equally spaced points within the interval [max(−5,min(x)),min(5,max(x))].
6. The point of maximum kernel density represents the point of maximum correspondence between Dam-fusion and Dam values; if both samples are correctly normalized this value should equal 0. We therefore normalize all Dam-fusion values by $1/\left(2^{\arg\max\left({\hat{f}}_{h}(x)\right)}\right)$.

In addition to ensuring correct normalization, a second important consideration is the reduction of background noise. Regions without specific methylation will have randomly distributed background counts that, when a ratio file is generated, will generate a large degree of noise. Such noise can potentially obscure peak detection. In order to mitigate this effect we add pseudocounts to both datasets. In order to maintain equivalence between replicates with differing numbers of reads (assuming that genome_bound_ ≪ genome_unbound)_ the number of pseudocounts added is proportional to the sequencing coverage, thus $c\frac{\text{reads}}{\text{bins}}$, where *c* is a constant. (Supplementary Table S1 for a comparison of gene calls with different read-depths). Adding pseudocounts increases the number and the total genomic coverage of detected peaks and increases the signal:noise ratio (Supplementary Figs S1–S4).

The combination of these two methods compares favorably with previously published microarray data [Fig. 1B (iv)] or DamID-seq data (Supplementary Figs S1–S4; Supplementary Fig. S5).

## 3 Implementation

The damidseq_pipeline software is implemented in Perl, and will process multiple single-end read sequencing files in FASTQ or BAM format. The pipeline can match sequencing adaptors to sample names, automatically identifies the Dam-only control, and performs alignment, read-length extension, normalization, background reduction and ratio file generation. (Supplementary Methods for details).

A large number of user-configurable options are provided, including the ability to adjust the normalization algorithm parameters, generate read-count normalized files and add a user-specified number of pseudocounts. Parameters specified on the command-line can be saved as defaults if the user desires.

The damidseq_pipeline software is open-source and freely available at http://owenjm.github.io/damidseq_pipeline. A detailed set of installation and usage instructions are provided at the above website, along with a small example dataset.

## Supplementary Material

Supplementary Data
