# Supplementary material for: damidseq_pipeline: an automated pipeline for processing DamID sequencing datasets
Source: Bioinformatics. 2015 Jun 25;31(20):3371–3. doi: 10.1093/bioinformatics/btv386 (PMC4595905; doi:10.1093/bioinformatics/btv386)
Supplement: Supplementary Data [file supp_31_20_3371__index.html]

damidseq pipeline: an automated pipeline for processing DamID sequencing datasets — damidseq\_pipeline: an automated pipeline for processing DamID sequencing datasets — damidseq\_pipeline: an automated pipeline for processing DamID sequencing datasets — Supplementary Data 

# damidseq\_pipeline: an automated pipeline for processing DamID sequencing datasets

## Supplementary Data

files

- Supplementary Data - pdf file
