# Supplementary material for: damidseq_pipeline: an automated pipeline for processing DamID sequencing datasets
Source: Bioinformatics. 2015 Jun 25;31(20):3371–3. doi: 10.1093/bioinformatics/btv386 (PMC4595905; doi:10.1093/bioinformatics/btv386)
Supplement: Supplementary Data [file supp_btv386_supplementary_material.pdf]

SUPPLEMENTARY MATERIAL

METHODS

Fly lines

Fly lines used were *UAS-LT3-NDam* and *UAS-LT3-NDam-RpII215* (Southall *et al.*, 2013), and *wor-GAL4* (Albertson *et al.*, 2004).

Targeted DamID

Targeted DamID was performed as previously described (Southall *et al.*, 2013), with the following modifications. Expression of the Targeted DamID proteins was driven using the neural stem cell-specific driver *worniu*-GAL4 in the presence of *tub*-GAL80ts and induced for 16 hrs at 29°C in third instar larvae. DNA was obtained from the anterior portions of forty torn larvae per sample.

Following the DamID procedure, samples were sonicated in a Diagenode Bioruptor to reduce the average DNA fragment size to 350bp, and DamID adaptors were removed via overnight *Sau3A*I digestion. The resulting DNA was purified using a PCR purification kit (Qiagen) and processed for Illumina sequencing using a Truseq-LT (Illumina) kit as per manufacturers instructions. 50bp single-end reads were obtained via a HiSeq 2500 (Illumina). Libraries were multiplexed such as to yield at least 20 million mappable reads per sample. Datasets were visualised using Gbrowse (Stein *et al.*, 2002).

Accession numbers

The RNA pol II DamID-seq raw and processed data have been deposited under NCBI GEO accession number GSE69184.

Previously published DamID-seq datasets

Dichaete DamID-seq datasets were obtained from Carl and Russell (2015) (Geo acc: GSE63333), using data from accession numbers GSM1545893 for Dam-only, and GSM1545886 and GSM1545887 for Dam-Dichaete. Dsx-1 male fat body DamID-seq data were obtained from (Clough *et al.*, 2014) (Geo acc: GSE27269), using data from GSM674125 for Dam-Dsx-1 and GSM674124 for Dam-only.

SRA datasets were converted to FASTQ via the SRA-tools software package (https://github.com/ncbi/sra-tools). Reads were trimmed to remove DamID adaptors, DamID adaptor dimers and low quality (<q30) ends using a custom Perl script (available upon request) before processing with the damidseq_pipeline software.

DNA binding profiles were visualised in IGV (Robinson *et al.*, 2011).

Software pipeline implementation

The software uses the following approach for processing NGS data in FASTQ format (processing of BAM files instead of FASTQ files starts at step 4).

1. Sequencing reads are aligned using the Bowtie 2 software package (Langmead and Salzberg, 2012).
2. The resulting SAM file reads with a Q-score > 30 are extended to the average length of sequenced fragments (300nt by default). This is achieved through modifying the SAM file, removing the sequence and quality lines (replacing with '*' in both instances) and setting the cigar string to the requisite length.
3. The modified SAM file is converted to a BAM file via the SAMtools software suite (Li *et al.*, 2009).
4. SAMtools is used to read the BAM file. Total readcounts are saved, chromosome names and sizes are determined from the header information and a hash of the genomic coverage is calculated for a user-specified bin size. For the *D. melanogaster* genome, we recommend using 75nt bins (the default setting) as a compromise between accuracy, coverage and file size; for the *Mus musculus* genome we recommend 500nt bins.
5. The binned coverage is reduced to GATC fragment resolution using a pre-built file containing the location of all GATC sites. Several pre-built files are provided online; files for other genomes can be built with a companion Perl script that is also provided.
6. Binned counts are normalised and pseudocounts are added as described.
7. Log_2(Dam-fusion/Dam) ratio files in either GFF or bedGraph format are generated for all samples excluding the Dam control, both at the GATC fragment resolution and (optionally) at the full resolution of the bins used to generate read counts.

Data analysis

Gene calls from RNA pol II DamID-seq data were performed using an R implementation of the gene-calling algorithm described in (Southall *et al.*, 2013) with the following modifications (R script available upon request). Briefly, for a defined set of log_2 thresholds in the range [0.1 ... 2], we take a random sample of the dataset over 50000 iterations, and determine the number of times the average occupancy is greater than the threshold for varying numbers of GATC fragments. Dividing the number of times the count exceeds the threshold by the number of iterations gives the FDR for this threshold/fragment number. Given that the relationship between GATC fragment number and log(FDR) for any threshold is linear, we determine the linear regression for each threshold. Since the relationship between the slope of each linear regression and the number of GATC fragments is also linear and the relationship between the number of GATC fragments and the intercept is quasi-linear, we obtain linear regressions for both these values. Knowing these final two regressions, it is possible to predict the FDR for a gene spanning any number of GATC fragments, with any average occupancy.

Peak calls were performed via the algorithm described in (Southall *et al.*, 2014) (Perl script available upon request).

Mean correlation between Dam-only and Dam-fusion protein binding was based on four DamID-seq datasets: RNA pol II in larval neuroblasts (this study); Dichaete in whole embryos (Carl and Russell, 2015); Dsx-1 in male fat body (Clough *et al.*, 2014); and Brm in larval neural stem cells (Marshall and Brand, unpublished).

All other analyses were performed using R (R Development Core Team, 2011).

SUPPLEMENTARY TABLES

Table S1. Effect of read depth on DamID-seq data. Random samples of the neuroblast RNA pol II DamID-seq dataset were compared for the signal:noise ratio, total peak coverage and the number of expressed genes called. Pseudocounts were added using the default value of 10 for *c*.

**Reads**	**SNR**[a]	**Total peak coverage (Mb)**	**Expressed genes (FDR < 0.01)**
1 x 10^6	0.91	10.60	1038
5 x 10^6	2.62	20.98	2140
10 x 10^6	3.09	24.27	2489
28 x 10^6	3.10	26.87	2427

[a]Signal:noise ratio calculated as (mean binding over peaks) / (SD of unbound regions)

Table S2. Effect of the changing the minimum quantile threshold on calculated normalisation values. Values were determined for RNA pol II in larval neural stem cells (this study), previously published data for the transcription factor Dsx-1 binding in male fat body (Clough *et al.*, 2014) and the chromatin factor Brm binding in larval neural stem cells (Marshall and Brand, unpublished). All Dam-fusion/Dam dataset pairs were reduced to equal numbers of readcounts before calculating the normalisation factor. Normalisation values are typically robust when the minimum quantile cutoff is set at between 0.4 (the software default) and 0.9.

	**Calculated normalisation value**		
**Min quantile cutoff**[a]	RNA pol II	Example transcription factor (Dsx-1)	Example chromatin factor (Brm)
0.1	2.99	1.26	2.94
0.2	3.83	1.50	2.63
0.3	3.75	1.46	2.90
0.4	3.63	1.40	2.54
0.5	3.66	1.42	2.48
0.6	3.65	1.47	2.43
0.7	3.71	1.51	2.41
0.8	3.72	1.54	2.46
0.9	3.70	1.59	2.49
1.0	4.29	2.00	2.54

[a]As set through the runtime –qscore1min option value

SUPPLEMENTARY FIGURES

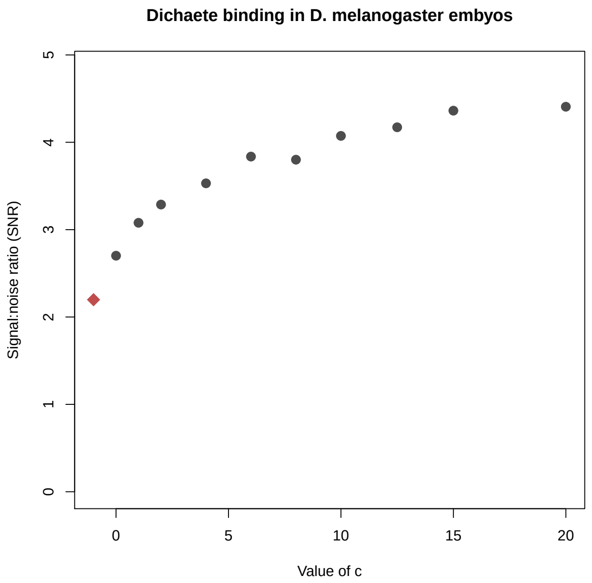

Figure S1. Effect of differing values of pseudocounts (as governed through differing values of *c*) on sample signal:noise ratio (SNR). SNR was calculated as (mean binding over peaks)/(SD of unbound regions). The red diamond represents the value obtained from previously published binding tracks (Carl and Russell, 2015).

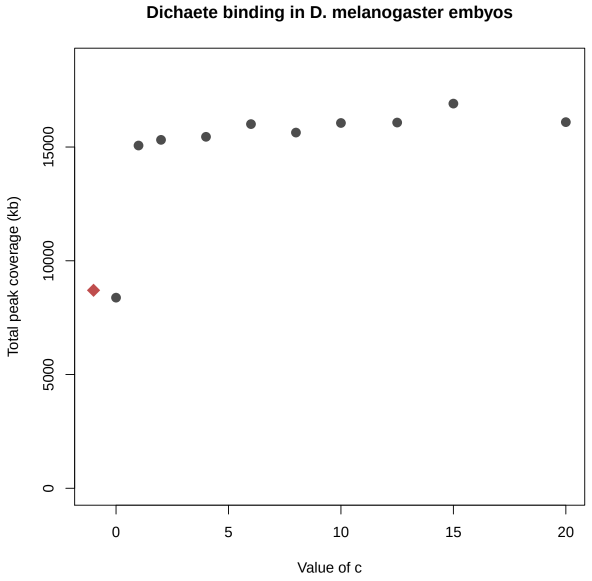

Figure S2. Effect of differing values of pseudocounts on total peak coverage (FDR < 0.01). The red diamond represents the value obtained from previously published binding tracks (Carl and Russell, 2015).

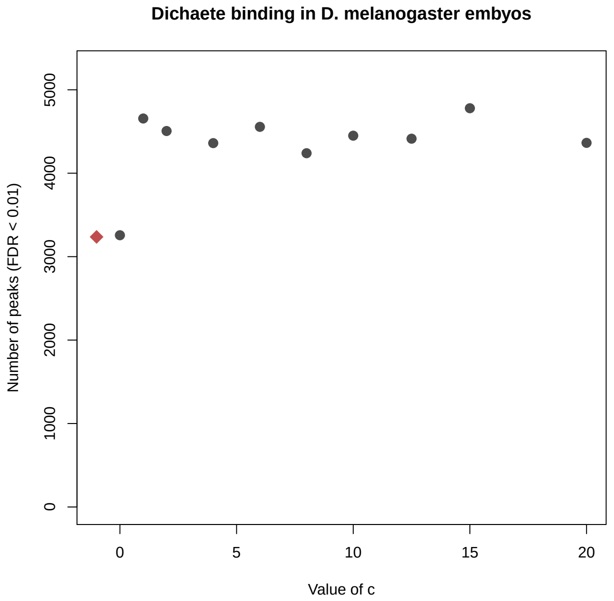

Figure S3. Effect of differing values of pseudocounts on the total number of detectable peaks with an FDR < 0.01. The red diamond represents the value obtained from previously published binding tracks (Carl and Russell, 2015).

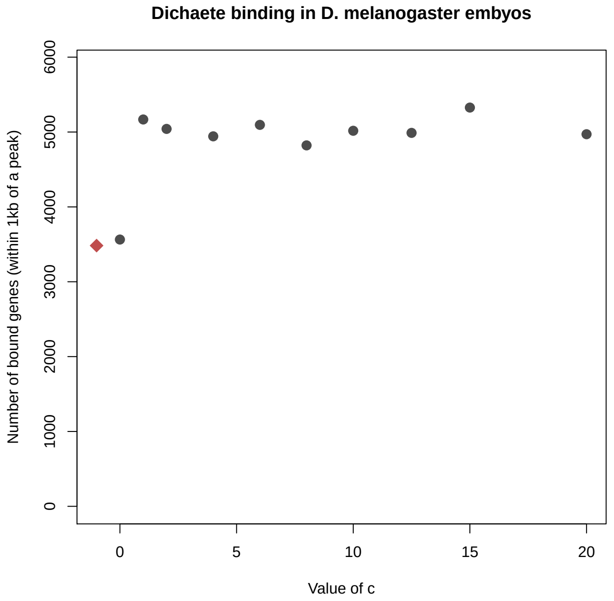

Figure S4. Effect of differing values of pseudocounts on the number of genes lying within 1kb of a peak (FDR < 0.01). The red diamond represents the value obtained from previously published binding tracks (Carl and Russell, 2015).

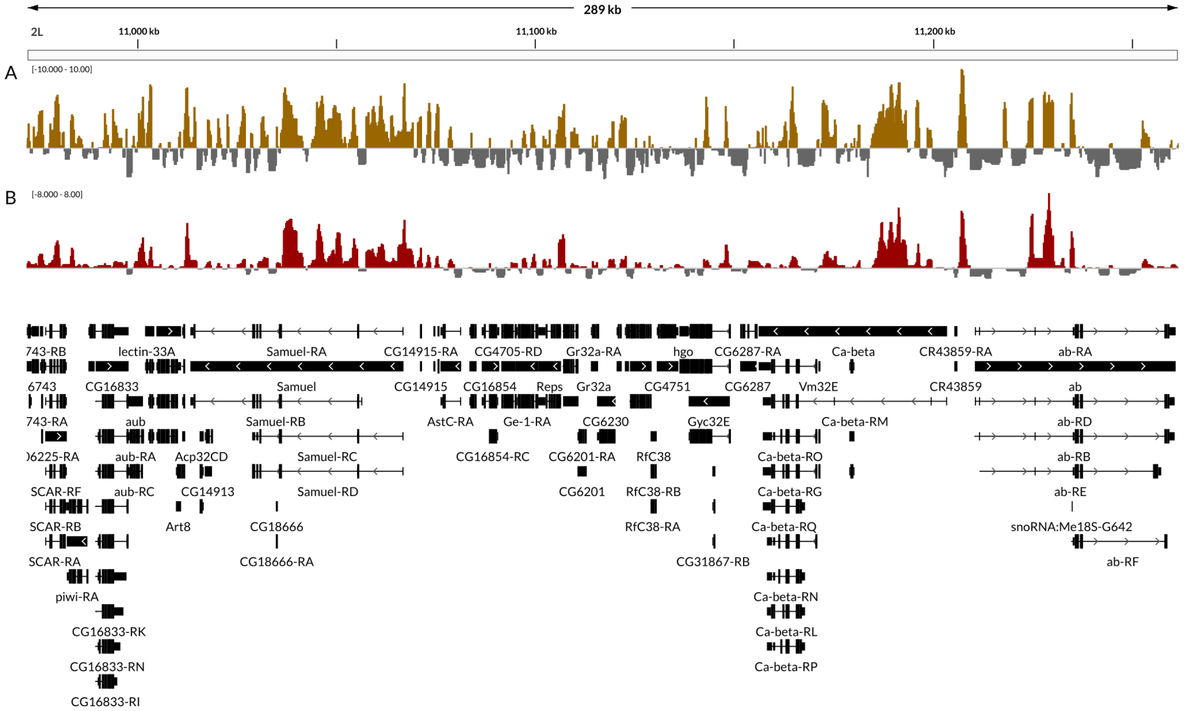

Figure S5. Processing of previously published DamID-seq data for Dicheate binding in *Drosophila melanogaster* embryos using the damidseq_pipeline software. (A) Published output from Carl and Russell (2015). (B) Re-processing the sequencing data using damidseq_pipeline removes the negative bias at unbound regions and generates binding profiles with reduced noise.

REFERENCES

Albertson, R., Chabu, C., Sheehan, A., and Doe, C. Q. (2004). Scribble protein domain mapping reveals a multistep localization mechanism and domains necessary for establishing cortical polarity. *J. Cell Sci.*, **117**(Pt 25), 6061–70.

Carl, S. H. and Russell, S. (2015). Common binding by redundant group B Sox proteins is evolutionarily conserved in Drosophila. *BMC Genomics*, **16**(1), 1–22.

Clough, E., Jimenez, E., Kim, Y.-A., Whitworth, C., Neville, M. C., Hempel, L. U., Pavlou, H. J., Chen, Z.-X., Sturgill, D., Dale, R. K., Smith, H. E., Przytycka, T. M., Goodwin, S. F., Van Doren, M., and Oliver, B. (2014). Sex- and Tissue-Specific Functions of Drosophila Doublesex Transcription Factor Target Genes. *Dev. Cell*, **31**(6), 761–773.

Langmead, B. and Salzberg, S. L. (2012). Fast gapped-read alignment with Bowtie 2. *Nat. Methods*, **9**(4), 357–9.

Li, H., Handsaker, B., Wysoker, A., Fennell, T., Ruan, J., Homer, N., Marth, G., Abecasis, G., and Durbin, R. (2009). The Sequence Alignment/Map format and SAMtools. *Bioinformatics*, **25**(16), 2078–9.

R Development Core Team (2011). R: A Language and Environment for Statistical Computing.

Robinson, J. T., Thorvaldsdóttir, H., Winckler, W., Guttman, M., Lander, E. S., Getz, G., and Mesirov, J. P. (2011). Integrative genomics viewer.

Southall, T. D., Gold, K. S., Egger, B., Davidson, C. M., Caygill, E. E., Marshall, O. J., and Brand, A. H. (2013). Cell-Type-Specific Profiling of Gene Expression and Chromatin Binding without Cell Isolation: Assaying RNA Pol II Occupancy in Neural Stem Cells. *Dev. Cell*, **26**(1), 101–12.

Southall, T. D., Davidson, C. M., Miller, C., Carr, A., and Brand, A. H. (2014). Dedifferentiation of Neurons Precedes Tumor Formation in lola Mutants. *Dev. Cell*, **28**(6), 685–696.

Stein, L. D., Mungall, C., Shu, S., Caudy, M., Mangone, M., Day, A., Nickerson, E., Stajich, J. E., Harris, T. W., Arva, A., and Lewis, S. (2002). The generic genome browser: a building block for a model organism system database. *Genome Res.*, **12**(10), 1599–610.
